# Ethics of Infection Control Measures for Carriers of Antimicrobial Drug–Resistant Organisms

**DOI:** 10.3201/eid2409.171644

**Published:** 2018-09

**Authors:** Babette Rump, Aura Timen, Marlies Hulscher, Marcel Verweij

**Affiliations:** The Netherlands National Institute for Public Health and the Environment, Bilthoven, the Netherlands (B. Rump, A. Timen);; Radboud University Medical Center, Nijmegen, the Netherlands (M. Hulscher);; Wageningen University, Wageningen, the Netherlands (M. Verweij)

**Keywords:** antimicrobial resistance, MDRO, ethics, carrier, control measures, infection control, bacteria, multidrug-resistant organisms, the Netherlands

## Abstract

Many countries have implemented infection control measures directed at carriers of multidrug-resistant organisms. To explore the ethical implications of these measures, we analyzed 227 consultations about multidrug resistance and compared them with the literature on communicable disease in general. We found that control measures aimed at carriers have a range of negative implications. Although moral dilemmas seem similar to those encountered while implementing control measures for other infectious diseases, 4 distinct features stand out for carriage of multidrug-resistant organisms: carriage presents itself as a state of being; carriage has limited relevance for the health of the carrier; carriage has little relevance outside healthcare settings; and antimicrobial resistance is a slowly evolving threat on which individual carriers have limited effect. These features are of ethical relevance because they influence the way we traditionally think about infectious disease control and urge us to pay more attention to the personal experience of the individual carrier.

Antimicrobial resistance (AMR) is one of the most serious health threats of the 21st century. It challenges effective treatment of infectious diseases, now and in the future. AMR may imply that infections that used to be relatively harmless will pose a severe threat to patients in the future (*1*). Many countries have implemented measures to control AMR, including proper use of antimicrobial drugs in humans, minimization of antimicrobial drug use in animals, and prevention of further transmission of resistant microbes within the healthcare system (*1*–*5*). AMR raises a range of ethical questions (*6*–*12*). We explored ethical issues that arise in relation to carriage of antimicrobial drug–resistant organisms (hereafter called carriage).

AMR control measures are directed at carriers. The types of control measures vary by microorganism and depend on resistance pattern, virulence, and mode of transmission. Measures can include control precautions taken during patient care, such as use of personal protective equipment; cleaning and disinfection of the care environment; dedicated single-patient use of rooms and equipment; eradication treatment, if applicable; and, in exceptional cases, exclusion of the carrier from work or joint facilities. The actual control measures recommended by health authorities vary greatly among countries. Countries in northern Europe, for instance, have implemented far-reaching infection control interventions that include preemptive use of contact precautions at the time of admission until the patient is proven culture negative and closure of hospital units to new admissions when applicable. Countries in southern Europe and North America follow a less aggressive approach, emphasizing contact precautions after detection of multidrug-resistant organisms (*1*–*4*).

Control measures may effectively control transmission of multidrug-resistant organisms, but negative effects on the health and well-being of carriers have been reported from countries that follow stringent multidrug-resistant organism policies and from countries that have a less aggressive approach (*13*–*16*). These negative effects make AMR control measures, apart from a technical and medical challenge, also an ethical issue. Our aim with this study was to examine the ethical context of multidrug-resistant organism carriage: what are the negative implications for carriers, and what is the ethical relevance?

## Methods

We analyzed 227 consultations/inquiries associated with multidrug-resistant organisms registered from January 1, 2008, through January 16, 2016, by the Centre for Infectious Disease Control in the Netherlands (Table 1; Figure). We looked for potentially negative implications on freedom, well-being, and other ethical values and assessed the respects in which the ethically relevant features of carriage differ from those of infectious disease in general. The Netherlands follows a strict multidrug-resistant organism search-and-destroy policy (Table 2) (*2*,*17*,*18*). Estimated prevalence rates for multidrug-resistant organisms in the Netherlands are low (Technical Appendix) (*2*,*19*–*21*).

**Table 1:** Detailed information from 227 consultations about antimicrobial-resistant organisms, Centre for Infectious Disease Control, Bilthoven, the Netherlands, January 1, 2008–January 16, 2016*

Characteristic	No. (%)
Type of multidrug-resistant organism	
Methicillin-resistant *Staphylococcus aureus*	177 (78)
Vancomycin-resistant *Enterococci*	18 (8)
Extended-spectrum β-lactamase	9 (4)
*Klebsiella pneumoniae* carbapenemase-producing *Enterobactericeae*	5 (2)
Unknown	18 (8)
Setting	
Long-term care facilities	61 (27)
Paramedical facilities	23 (10)
Home-care facilities	14 (6)
Rehabilitation centers	5 (2)
Carriage among healthcare workers	50 (22)
Social interaction of healthcare workers	32 (14)
Other	42 (19)

*In the Netherlands, 25 regional Public Health Services (PHS) are in charge of communicable disease control. Healthcare institutions such as hospitals and nursing homes have a responsibility to detect, monitor, and control outbreaks within their facility and report these to the PHS. The PHS assists healthcare institutions and professionals and provides advice on the basis of national guidelines. In turn, the Centre for Infectious Disease Control of the National Institute of Public Health and the Environment (RIVM) acts as national public health authority; it develops and publishes national guidelines and offers support in outbreak management including a 24-hour consultation helpdesk for PHS and other health professionals. The center is consulted by PHS professionals >1,000 times/y about a variety of cases of notifiable diseases, outbreaks, and incidents that occur in the community (*15*,*17*,*18*). Since 2008, all consultations have been anonymously registered in a database. During the 8-year study period, RIVM registered 227 consultations associated with carriage of multidrug-resistant organisms that needed national guidance.

**Figure:**
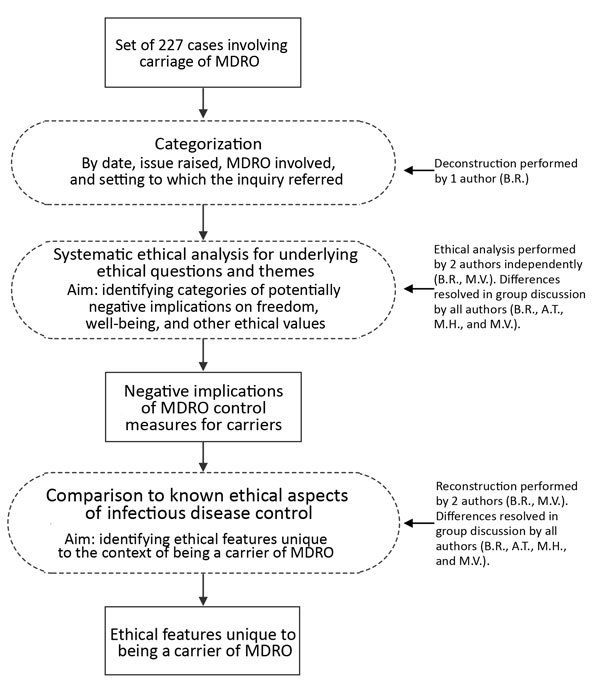
Methods used in study of ethics of infection control measures for carriers of antimicrobial-resistant organisms, the Netherlands, January 1, 2008–January 16, 2016. MDRO, multidrug-resistant organism.

**Table 2:** Indications for routine screening for multidrug-resistant organisms, the Netherlands*

Healthcare setting	Indication†
Hospital	Patients at high risk of carrying an MDRO (e.g., patients transferred from a hospital in a foreign country or patients working in animal husbandry)
	Patients at high risk of acquiring infection with an MDRO
	Patients with signs of clinical infection with an MDRO
	Patients for whom empiric treatment failed
	Patients with recurrent infection
	Family members of hospital patient known to carry an MDRO
	Personnel with unprotected exposure to a person known to carry MRSA
General practice	Patients for whom empiric treatment failed
	Patients with recurrent infection
Nursing home/care facility	Patients for whom empiric treatment failed
	Patients with recurrent infection
	Patient with unprotected exposure (e.g., shared a room, shared medical equipment) to a person with MRSA or carbapenemase-producing *Enterobacteriaceae*
	Personnel with unprotected exposure to a person known to carry MRSA
Home	Personnel with unprotected exposure to a person known to carry MRSA

*MDRO, multidrug-resistant organism; MRSA, methicillin-resistant *Staphylococcus aureus*. †As advised by the Werkgroep Infectie Preventie guideline on measures against transmission of highly resistant microorganisms in hospitals (*2*,*19*,*20*).

## Results

### Negative Implications of Control Measures for Carriers

#### Problems with Access to Healthcare

A clear implication of AMR control measures involves problems with access to healthcare. During their consultations, several carriers asked about being faced with postponement of planned surgery, about cancellation of admission to rehabilitation, and about being denied access to dental clinics. A nursing home, for instance, wanted to deprioritize a person at the top of the waiting list because this person was carrying a multidrug-resistant organism. A medical daycare center refused to admit a child because of persistent carriage.

#### Restrictions within Healthcare Facilities

Another distinct implication of AMR control measures involves restrictions within healthcare facilities. Several consultations involved questions about carriers of methicillin-resistant *Staphylococcus aureus* (MRSA) in care facilities in which elderly carriers were banned from organized social activities or not allowed to dine at the same table with fellow residents. In medical daycare facilities, children who were carriers were banned from group activities or kept away from their peers, and in a psychiatric institution, a group of patients was placed in a closed ward because of carriage. Other inquiries concerned privileges that carriers received; for instance, carriers in nursing homes were allocated a single room or a private bathroom.

#### Negative Implications for Daily Life

The control measures also affected daily life. One inquiry concerned a MRSA-positive child who faced restrictions after returning to school because a classmate was a cystic fibrosis patient for whom acquiring a MRSA infection would constitute a health risk. Another inquiry was about adoption of a child with special health needs; the family had already adopted their first child with a previous diagnosis of persistent MRSA carriage, and they hesitated to adopt a second child because the MRSA would most likely be transmitted to that child, bringing extra MRSA-associated health risks. Also, parents of a healthy MRSA toddler were confronted with a daycare center caregiver who refused to attend to their child for fear of transmitting MRSA to her newborn baby at home. Some inquiries concerned interaction with animals; for instance, a family struggled with persistent MRSA carriage and 1 of their children was denied access to a medical daycare center. They were advised to relocate or abandon their cats, which were thought to be the source of reinfection.

#### Negative Implications for Carriers Who Work in Healthcare

Control measures can also have negative implications for those who work in healthcare. We found cases of healthcare workers (HCWs) who were restricted at work, banned from work, and faced income loss. For example, a nurse who was a carrier was assigned administrative tasks instead of patient care, thereby missing out on the substantial financial benefits that come along with performing patient care during night and weekend shifts. A temporary employee’s contract was not renewed because of past carriage, and a fifth-year medical student discontinued training because of a chronic MRSA infection. HCWs were also pressed to cooperate with testing and treatment. A temporary healthcare employee was asked to show proof of being MRSA negative, and MRSA-positive nurses were pressed to cooperate with intensive eradication treatment consisting of daily scrubbing of the skin and taking of oral antimicrobial drugs. In several instances, MRSA-negative HCWs were excluded from healthcare work because in their private life they cared for a MRSA-positive child or parent.

#### Negative Implications for Close Contacts of HCWs

Infection control measures for HCWs can also affect their family members and other contacts. For example, HCWs with MRSA were asked to disclose the names of their close contacts outside the hospital. Contacts needed to cooperate with MRSA screening and, if test results were positive, undergo eradication treatment. In some instances, such measures had far-reaching consequences for family members. For instance, in a single-income household, young children were subjected to very intensive MRSA eradication in order for the main breadwinner to be able to secure employment. In another case, contact screening started by the employer of a nurse who was a carrier included screening of the nurse’s children. One child was physically handicapped and visited a medical daycare center. When results indicated that he was a carrier, he was denied access to this medical daycare center for several months.

The negative implications for carriers of multidrug-resistant organisms were not only defined by the outcome of the control measures advised in the policies but also were further enhanced by focus on collective benefits with less emphasis on harm for carriers (*1*) and by strong concerns with communication and disclosure when applying the policy (*2*). Several inquiries resulted in implementation of control measures that were more stringent than those prescribed by national policies.

#### Negative Implications because of Overemphasis on Collective Benefits

Inquiries reflected a strong focus on the benefits of AMR control measures and ignoring of the potential harm for carriers. Several inquiries reported control measures that went beyond the already stringent national policies. For example, in 2015–2016, a large influx of war refugees from Syria to the Netherlands caused some hospitals to demand that their employees refrain from volunteer work with refugees outside their working hours because of the possibility that they could be exposed to a multidrug-resistant organism by doing such work. In addition, a pig farmer who had undergone heart valve surgery was advised not to go back to work on the farm because of the small risk of contracting livestock-associated MRSA, which would make follow-up visits more complicated to schedule for the hospital.

#### Negative Implications because of Concerns about Communication and Disclosure

Some inquiries reflected outcomes that were motivated by concerns about disclosure and communication rather than actual risk for transmission of the multidrug-resistant organisms. For instance, a MRSA-positive child was not allowed access to a medical daycare facility, not because of the risk to other children, which was considered to be small, but because the facility felt an obligation to inform all other parents. The parents of the carrier, however, insisted on nondisclosure for fear of stigma. Another inquiry concerned a nurse who lived on a livestock farm and was therefore at high risk of contracting MRSA, a risk that was well-known and had been accepted by her employer for years. When, by accident, the nurse was screened and carriage was confirmed, she was no longer allowed to work at that facility. This response was not motivated by the risk for transmission—the employer acknowledged that she presumably had been carrying MRSA for a long time and had never caused an outbreak—but because the institution was concerned about the consequences should MRSA carriage of a hospital employee become public.

### Ethical Features Unique to Being a Multidrug-Resistant Organism Carrier

Inquiries concerned questions about AMR control measures that primarily aimed to reduce further transmission of antimicrobial-resistant pathogens. In doing so, these measures resulted in negative implications that raised moral dilemmas.

In the inquiries explored, the exact nature of the moral dilemmas remained implicit. However, for almost all cases, it could be assumed that the control measures had negative effects on the carrier’s well-being, autonomy, and (health-associated) justice. Well-being was affected because carriers were limited in their opportunities to work or to engage in social contacts. Autonomy may have been at stake when carriers were requested to disclose their medical condition or when they were pressed to undergo tests and eradication therapy they might have preferred to avoid. Their sense of dignity may have been affected when carriers were stigmatized because of their condition. The various implications also seemed to be involve injustices: health inequity if carriers were excluded from certain medical treatment or faced a delay in care, and social injustice if they were excluded from (the benefits of) going to work.

Although challenging, the moral dilemmas at hand—and the values at stake—seem not fundamentally different from dilemmas that arise in infectious disease control in general (*22*–*25*). Health equity issues, for instance, occur in many contexts of infectious disease control. In Europe, while the 2014–2015 Ebola outbreak was occurring in West Africa, persons suspected of having Ebola virus disease were banned from hospital emergency rooms (*26*). Often at the heart of outbreak management are quarantine, isolation, and social distancing measures, which clearly involve tensions with respect to autonomy and deprive persons from contact with their loved ones and otherwise undermine their quality of life (*25*). Restrictions to healthcare staff (e.g., a surgeon who seems to be a hepatitis B virus carrier) are well-accepted ways to prevent bloodborne nosocomial infections (*27*). However, 4 differences stood out, suggesting that there is something ethically noteworthy about carriage of multidrug-resistant organisms.

#### Relevance of Carriage for the Carriers

Patients in this study were asymptomatic carriers for whom carriage did not affect their health. Some might have had other health conditions, but they were not ill from the drug-resistant microorganism they carried. Thus, carriage differs from most communicable diseases, in which the health of the persons carrying the microorganism is threatened or affected by the infection. Ebola virus infection, for instance, forms an acute threat to the health of the patient, who is in immediate need of treatment and medication while threatening the health of others, including health personnel. Other infectious diseases can also involve asymptomatic carriage; moreover, multidrug-resistant organisms can certainly also cause infections and thus illness. In fact, the proactive screening and preemptive use of control measures that are common in the Netherlands probably caused an overrepresentation of inquiries concerning these “carriers without multidrug-resistant organism infection” (*2*,*19*,*20*). What remains ethically noteworthy and relevant for preemptive and reactive AMR control strategies is that, although all carriers are at risk for their carriage resulting in clinical infection, multidrug-resistant organisms primarily threaten a specific subgroup of vulnerable patients in hospital settings. The extent to which multidrug-resistant organisms contribute to death has been debated and seems to remain limited to those with severe illness and concurrent conditions (*28*–*30*). Studies addressing multidrug-resistant gram-negative infections, for instance, show substantial diversity in the outcomes. It can be concluded that mortality rates are higher among those infected by multidrug-resistant gram-negative bacteria; however, concurrent conditions and severity scores are more commonly identified as predictors of death (*28*–*30*). From a broader public health perspective, the health threat of multidrug-resistant organism carriage thus appears limited.

#### Healthcare-Associated Relevance

A noteworthy finding is that carriage became relevant almost exclusively in healthcare-associated settings. In schoolchildren, for example, carriage was problematic because a classmate had a chronic illness and needed regular hospital checkups. A MRSA-positive family member is only problematic in the context of work in healthcare. Again, most outbreaks of infectious diseases are problematic within healthcare-associated settings, because these outbreaks lead to high morbidity and mortality rates, putting pressure on limited resources and putting HCWs in direct danger of contracting disease. Control measures for most communicable diseases therefore aim to regulate these threats (*25*). Outbreaks of multidrug-resistant organism infections, however, do not cause high morbidity and mortality rates (*21*,*28*–*30*). Public health measures aim to prevent introduction and further transmission of multidrug-resistant organisms in (some) healthcare-associated settings (*2*). Whether a carrier is subject to control measures does not depend on the severity of the pathogen but only on the likelihood that the resistant pathogen will be transmitted to a healthcare setting where vulnerable patients are cared for.

#### Multidrug-Resistant Organism Carriage as a State of Being

A salient feature of the inquiries was that carriage could last for a long time, making implementation of control measures even more burdensome. Some persons were colonized for such a long period, some even starting at birth, that it could be argued that the resistant microorganism was now part of their regular flora. The inquiries showed that, after a person receives a diagnosis of being a carrier, the label persists. It was often very difficult to eradicate the bacteria; moreover, there was no standard for determining whether a person was no longer a carrier. From an ethical perspective, persistence is particularly salient because inevitably, within the open population but also in healthcare settings, there will be a substantial group of unidentified asymptomatic carriers. Therefore, the severe restrictions faced by known carriers may not only be burdensome and stigmatizing but may also be considered unfair.

#### The Carrier as a Nondefining Factor in a Slowly Evolving Threat

In all cases analyzed for this study, the individual carrier was a possible link in the chain of transmission but certainly was not a central factor in the emergence and spread of multidrug-resistant organisms. The long-term clinical effect of multidrug-resistant organisms may be high, but it was not obvious that imposing restrictions, either preemptive or reactive, on individual carriers played a crucial role in controlling and mitigating that effect. The immediate threat posed by individual carriers was limited, certainly if compared with the role of conditions caused by other microorganisms, such as Ebola virus disease or meningococcal meningitis, for which devastating effects become evident in days, weeks, or months (*25*).

## Discussion

We have shown how multidrug-resistant organism control measures undermine the well-being of asymptomatic carriers. Although set in a country at the highest end of the spectrum with regard to strict AMR control measures, this finding is relevant to countries with all types of policies. The unique ethical features of multidrug-resistant organism carriage challenge the way we think about infectious disease control.

Traditionally, epidemics have been portrayed as an enemy attack of foreign microbes on human life, describing the carrier as “patient” or “victim” (*25*,*31*). However, multidrug-resistant organism carriers are not ill from carriage and can remain colonized for a long time. Any role as victim results more from the control measures than from the pathogen.

AMR control measures that may seem reasonable at first can easily lead to stigmatization. Stigma is defined as a social process characterized by exclusion, rejection, or blame resulting from experience, perception, or anticipation of adverse social judgments (*32*). In infectious disease control, the line between reasonable precaution measures and stigmatization has always been thin (*32*–*35*), but when carriage resembles a state of being, with limited relevance outside healthcare, the line also becomes vague and ambiguous (*33*).

Still, the dilemma of multidrug-resistant organism carriage represents one of the universal ethical challenges of public health: balancing the protection of the public while respecting individual well-being. Various public health ethics frameworks to guide decision-making have been suggested in this trade-off (*24*,*36*–*38*). Those frameworks have in common that they, explicitly or implicitly, call for clarity about the goals of a program and evaluation of effectiveness and proportionality. Such clarity is indeed valuable, but for multidrug-resistant organism control measures, the ultimate goals are not obvious. Of course, control measures are meant to control further spread of AMR, yet at the same time, overall mortality rates caused by multidrug-resistant organisms are still low and limited to vulnerable patients. Moreover, AMR is not a single epidemic; rather, it is a complex problem that slowly evolves and continually reemerges. Types of microorganisms displaying resistance and resistance mechanisms are constantly evolving. How AMR will emerge and what implications it will have in the next decades has yet to be determined (*39*). Although the control of AMR is of utmost importance, it is not obvious that strict control measures imposed on carriers will make a big difference in the overall objective.

AMR resembles a “wicked problem,” a policy challenge that is not solvable by traditional policy instruments and to which no singular solution exists (*8*,*9*). Our analysis shows that control measures can be highly burdensome to carriers and that the magnitude of burden depends largely on the carrier’s personal situation. Tailoring control measures to individual carriers’ needs and values may therefore offer a way to deal with the wicked complexity.

Rather than asking whether it is justified to impose strict control measures to prevent antimicrobial resistance transmission from carriers, we propose asking, “How can we best care for this person’s carriage and well-being in ways that do not imply unacceptable risk (for transmission) for other patients?” This question essentially takes an individualistic and contextual approach, acknowledging that different carriers can have different needs and values. For instance, some carriers enjoy the privacy that comes with isolation, many dislike the solitude, and others are most concerned about the quality of care and are relatively indifferent to isolation.

The question touches on the idea of patient-centered care, which involves caring for patients (and their families) in ways that are meaningful and valuable to each patient (*40*). At the same time, the problem goes beyond the scope of healthcare. Often the primary needs of carriers are not so much healthcare needs but rather are protection of the possibility that they can live a good life according to their own personal values. From this perspective, frameworks that use a rich account of quality of life may be helpful for evaluating the justice of control measures (*41*–*45*).

The question also requires critical reflection on the assessment of the risks of possible transmission of AMR to others in this specific context, ruling out that control measures imposed on individual persons are (implicitly) justified by appeal to the general (long-term) public health threat of AMR. A specific level of risk may be acceptable in a hospital in a region where baseline prevalence is high yet problematic in one where prevalence is low.

Especially when strict control measures are justified, an individualistic approach can help lower the individual burden. A nurse carrying multidrug-resistant organisms can be given other tasks instead of being sent home, some carriers could be compensated for financial consequences, and others could be helped by provision of childcare or extra support at home. Relieving the burdens of control measures on carriers will often come with financial costs for society or healthcare institutions, but it would be unreasonable if burdens of public health measures are borne by carriers individually.

In summary, AMR is one of the most severe threats of this century and control measures are needed; however, these measures are highly burdensome for carriers and of only limited benefit to the overall problem. Tailoring measures to personal needs and values of carriers may offer a new way to prevent carriers’ transmission of multidrug-resistant organisms while minimizing compromises to their well-being.

Technical Appendix Estimated prevalence of multidrug-resistant organisms in the Netherlands, January 1, 2008–January 16, 2016.
