# Supplementary material for: Ethics of Infection Control Measures for Carriers of Antimicrobial Drug–Resistant Organisms
Source: Emerg Infect Dis. 2018 Sep;24(9):1609–16. doi: 10.3201/eid2409.171644 (PMC6106419; doi:10.3201/eid2409.171644)
Supplement: Technical Appendix — Estimated prevalence of multidrug-resistant organisms in the Netherlands, January 1, 2008–January 16, 2016. [file 17-1644-Techapp-s1.pdf]

Article DOI: https://doi.org/10.3201/eid2409.171644

Ethics of Infection Control Measures for Carriers of Antimicrobial Drug–Resistant Organisms

Technical Appendix

Estimated MDRO prevalence in the Netherlands

Trends in antimicrobial resistance (AMR) in the Netherlands are monitored through the national AMR surveillance system (ISIS-AR) (*1*–*3*). The surveillances system uses the routine antibiotic susceptibility testing data from microbiology laboratories. These samples result from screening based on the Dutch screening policy which defines which categories of patients belong to the risk groups and which contacts should be included in the contact tracing during an outbreak. Results are published annually in the NethMap/MARAN-report (*1*–*3*).

The Netherlands has one of the lowest prevalence of multidrug-resistant organisms (MDRO) compared to other countries in Europe. For 2016 prevalences of MDRO in the Netherlands were estimated as follows:

- The percentage of Carbapenem-Resistant *Enterobacteriaceae* (CRE) E. *Coli* and K. *Pneumoniae* isolates was 0.01% and 0.15% respectively
- The percentage of Vancomycin-Resistant *Enterococci* (VRE) isolates varied between 0.2% in the outpatient departments to 0.8% in intensive care units
- The percentage of invasive Methicillin-Susceptible *Staphylococcus aureus* (MRSA) isolates was 1% (2,5% in samples from general practitioners patients)
- The percentage of Extended-spectrum β-lactamase–producing *Enterobacteriacea*e varied between 3.1% in general practitioners practices to 8.4% in intensive care units

References

1. Altorf-van der Kuil W, Schoffelen AF, de Greeff SC, Thijsen SF, Alblas HJ, Notermans DW, et al.; The National AMR Surveillance Study Group. National laboratory-based surveillance system for antimicrobial resistance: a successful tool to support the control of antimicrobial resistance in the Netherlands. Euro Surveill. 2017;22. PubMed http://dx.doi.org/10.2807/1560-7917.ES.2017.22.46.17-00062

2. de Greeff SC, Mouton JW. NethMap 2017: consumption of antimicrobial agents and antimicrobial resistance among medically important bacteria in the Netherlands/MARAN 2017: monitoring of antimicrobial resistance and antibiotic usage in animals in the Netherlands in 2016 [cited 2018 Jun 26]. https://www.rivm.nl/Documenten_en_publicaties/Wetenschappelijk/Rapporten/2017/Juni/NethMap_2017_Consumption_of_antimicrobial_agents_and_antimicrobial_resistance_among_medically_important_bacteria_in_the_Netherlands_MARAN_2017_Monitoring_of_antimicrobial_resistance_and_antibiotic_usage_in_animals_in_the_Netherlands_in_2016

3. The European Centre for Disease Prevention and Control. Data from the ECDC Surveillance Atlas—antimicrobial resistance [cited 2018 Feb 25]. https://ecdc.europa.eu/en/antimicrobial-resistance/surveillance-and-disease-data/data-ecdc
